# Supplementary material for: Are carbon emissions trading and green financial instruments synergistic? -Comprehensive quantitative research based on content analysis
Source: PLoS One. 2024 Mar 7;19(3):e0298601. doi: 10.1371/journal.pone.0298601 (PMC10919601; doi:10.1371/journal.pone.0298601)
Supplement: S1 Data — (ZIP) [file pone.0298601.s001.zip › Data/地级市Z_F工作报告.zip!/║╙▒▒/╨╧╠¿╩╨/└┤╫╘╣1⁄2╓┌║┼í╛┬φ┐╦╩2╛▌═°í┐_2009.docx]

各位代表：

　　现在，我代表邢台市人民政府向大会作工作报告，请予审议，并请市政协委员和列席会议的同志提出意见。

　　一、2008年工作回顾

　　刚刚过去的一年是邢台历史上极不寻常、极不平凡的一年。面对复杂多变的经济形势和诸多不利因素，我们在省委、省政府和市委的正确领导下，认真贯彻落实科学发展观，解放思想，真抓实干，积极应对各种困难和挑战，全力推进经济社会平稳较快发展，较好地完成了市十三届人大一次会议确定的目标任务。

　　——经济总体保持平稳较快发展。初步预计，全市生产总值完成989亿元，同比增长10%左右。全部财政收入完成102.3亿元，增长20.1%；其中地方一般预算收入完成36.9亿元，增长17.7%。农业经济加快发展。认真贯彻温家宝总理到邢台视察指示精神，全面落实支农惠农政策。粮食再获丰收，单产、总产均创历史新高，总产412万吨，增长4.6%。棉花总产21.8万吨，增长0.9%。畜牧、蔬菜、林果三大产业占大农业比重达到58%，同比提高3个百分点。产业化经营率达到60%，同比提高3个百分点。工业经济保持增长。全市规模以上工业增加值完成354亿元，增长11.5%；实现利税157亿元，其中利润106亿元，分别增长30%、20%。第三产业活力增强。全年完成增加值276亿元，增长13.3%，高于全市生产总值增幅3.3个百分点；全社会消费品零售总额完成330亿元，增长21%，同比提高3.2个百分点。民营经济不断壮大。全市民营经济完成增加值和实缴税金分别为643亿元和50.6亿元，占全市生产总值和全部财政收入的65%左右和49.5%左右。

　　——结构调整和发展方式转变取得成效。积极推进了重点项目建设。预计全社会固定资产投资完成603.5亿元，增长16%；其中城镇固定资产投资完成431.9亿元，增长21%。全市144个重点在建项目完成投资230亿元，50个项目竣工投产；省政府调度我市的76个重点建设项目完成投资199.5亿元，占年度投资计划的107%。建滔煤化工、今麦郎方便面及饮品等2个项目被列为河北省第三批重点产业支撑项目。“三新三特”产业加快发展。中钢邢机技改、德龙精品轧辊一期、晶龙集团250兆瓦太阳能电池等项目建成投产或开工建设；德龙、邢钢、晶龙等企业销售收入超百亿元；建设和完善了清河·濮院羊绒制品、沙河金百家等专业市场和产业园区，促进了产业聚集。扎实推进了节能减排工作。列入省考核的“双三十”重点县和重点企业均完成或超额完成了年度节能减排目标；列入市“双三十”的30个重点工业集中区和30家重点企业节能减排工作取得阶段性成效。关停兴泰发电4号、7号两台共44万千瓦火电机组，淘汰不符合产业政策的10台钢铁企业小型烧结机，关闭72条玻璃企业落后生产线，拆除44座水泥企业机立窑，爆破100座土石灰窑，实施了一批高炉煤气回收、余热余压利用、尾气发电等节能项目。预计全市万元GDP能耗降低5%，COD和二氧化硫排放量分别下降7%和10%；市区空气质量二级及好于二级天数列全省第3位，比去年增加14天，超额完成年初目标。

　　——城镇建设取得新突破。预计全市城镇化率达到39.05%，提高近1个百分点。城市总体规划通过省政府审定，确定了城镇总体框架。完成了三年大变样规划，一批重点工程进展顺利。七里河综合治理工程完成投资5.5亿元，玉带桥、守敬南路大桥等工程正在加紧建设；对团结大街、邢州路、泉北大街等街道进行了改造和整治；知春园、金牛中兴广场等游园广场建成或加快实施。完成了26条小街巷的亮化改造，市区主要街道亮化率达到95%以上；拆墙透绿、“穿衣戴帽”等工程全面启动，新增绿化面积49.6万平方米。全市拆除违章建筑、超期临建和有碍观瞻的低矮、破陋建筑575万平方米，其中市区305万平方米，超额完成全年任务目标。供气供热完成投资2亿多元，新增燃气用户1.1万户，发展集中供热面积215万平方米。邢台市被命名为全国首座“太阳能建筑城”。各县（市）城镇建设了一批基础设施工程，南宫市、清河县、宁晋县污水处理厂建成并投入运行。交通建设完成投资22亿元，大广高速公路开工建设，对106、107国道等干线公路实施了改造，建设农村公路876公里。

　　——改革开放步伐加快。为拉大中心城市框架、加速中心城市发展，科学调整了市对邢台县部分管理体制。成立了市城市管理行政执法局，理顺了城市管理体制。市属企业改革取得新进展，河北古顺酒业有限公司、市床单厂等破产企业职工安置工作全面展开，市薄板厂、市日化厂等7家企业国有产权实现整体转让，河北邢台电缆有限公司等5家市属企业退市进区前期工作正在有序进行。加大招商引资力度，同世界500强日本矢崎株式会社、美国AP公司达成初步合作意向。东莞市商业银行注资7500万元入股，与我市商业银行开展合作。积极促进政银企合作，组织117家中小企业与驻市各金融机构达成总额度56亿元的投融资意向，中国建设银行河北分行将在今后两年为我市提供200亿元金融支持。启动了资源、资产、资金、资本“四资”整合平台筹建工作。预计全年实际利用外资2亿美元；引进内资97亿元，增长30%。全年进出口总值17.5亿美元，增长60%，其中出口总值10.3亿美元，增长30%。

　　——人民生活继续改善。预计城镇居民人均可支配收入12100元，增长14.4%；农民人均纯收入4175元，增长7.6%。就业和社会保障水平得到提高，城镇新增就业4.4万人，城镇下岗失业人员实现再就业1.7万人，全市农村劳动力向非农产业转移22.3万人（次），均超额完成年初目标；城镇登记失业率4%，低于省控目标；养老、医疗、失业、工伤、生育五大保险新增参保16万人；农村低保扩大了覆盖面，人年均保障标准提高到926元；城镇低保对象人月均保障标准提高到214元；全市28所星级敬老院全部开工建设，其中6所建成投入使用，2.5万名五保对象得到有效保障。加大扶贫投入力度，159个贫困村1.7万户贫困家庭得到资金支持，带动了8.2万人增收；扶贫开发重点县农民人均纯收入增幅高于全市平均水平。在全市推广联村供水模式，解决了25万农村人口的饮水安全问题。群众居住条件得到改善，市区经济适用住房货币补贴标准由每户2.5万元提高到3万元，补贴户数达到1000户。对廉租住房租赁补贴家庭实现了应保尽保。

　　——社会事业全面进步。教育工作整体推进，农村义务教育阶段学生的教科书全部免费发放，家庭经济困难寄宿生生活费资助面由上年的10.9%扩大到22.6%，城市义务教育阶段学生也享受到免学杂费政策。完成了2336所农村中小学取暖设施改造和85所中小学校舍改造，消除危房面积1万平方米，新建校舍12万平方米，均完成年初目标。邢台职业技术学院、河北机电职业技术学院、市农业学校等3所学校被列为省级职教集团牵头单位，占全省的五分之一。积极推进科技创新，9项科技成果获得河北省科技进步奖；科研院所创新能力得到提升，科技成果转化步伐加快；与河北理工大学、河北工业大学、河北农业大学等高校签订了合作协议。卫生体系更加健全，新型农村合作医疗实现了全覆盖，参合率达到86%，比上年提高了10个百分点；大力实施以“一改善、两提高”为主要内容的“春雨工程”，乡镇卫生院和社区卫生服务工作得到加强；切实做好三鹿牌婴幼儿配方奶粉重大安全事故应急处置工作，就诊患儿得到有效救治。计划生育后进村整治成效显著，保持了低生育水平。深入开展了三项集中整治活动，加强对食品药品质量安全的监督管理，安全生产形势保持稳定。年初，积极支持南方抗击雨雪冰冻灾害。“5·12”汶川特大地震发生后，大力支援四川灾区，在对口援建中创造了到达时间最早、建设速度最快、交付使用最先的“邢台速度”。全力做好奥运安保、信访稳定和社会治安等各项工作，保持了社会稳定。不断丰富群众文化生活，举办了纪念改革开放三十周年系列活动。国防动员和“双拥”共建深入推进，军政、军民团结的局面进一步巩固。国土资源、体育广电、民族宗教、外事侨务、妇女儿童、老龄、慈善、残疾人、应急管理、人防、气象等各项工作也都取得了新成绩。

　　——政府职能加快转变。强化社会管理和公共服务，着力优化发展环境。针对新食尚公司反映的发展环境问题，对行政审批、办事效率、工作作风等进行了深入整改，取得了初步成效。市级行政许可事项减少126项，对不符合现行法律法规或发展实际的21个规范性文件予以废止。积极推进行政权力公开透明运行，按要求对政府信息进行了公开。深入开展了廉政建设和反腐败斗争。规范行政行为，依法行政水平不断提高。自觉接受人大及其常委会和政协的监督，全年共办理市人大代表建议228件、市政协提案597件，按时办复率均为100%，主动听取各方面意见，接受社会各界的监督。

　　各位代表，过去一年来各项工作取得的成绩来之不易，这是省委、省政府和市委正确领导的结果，是市人大、市政协和各民主党派监督支持的结果，是全市人民和衷共济、辛勤劳动的结果。在此，我代表邢台市人民政府，向全市广大工人、农民、知识分子、各级干部和社会各阶层的建设者，向人大代表、政协委员，向各民主党派、工商联、无党派人士和人民团体，向各位老领导、老同志，向驻邢部队、武警官兵和政法干警，向中省直驻邢各单位，向所有关心和支持邢台发展的海内外朋友，致以崇高的敬意和衷心的感谢！

　　在肯定成绩的同时，我们也清醒地认识到，我市经济社会发展和政府工作中还存在一些矛盾和问题。一是综合经济实力弱，发展水平不高。与全省各设区市和中原协作区十三市相比，主要经济指标不但总量小，而且增速低，与先进地区的差距有越拉越大的趋势。受金融危机影响，去年下半年以来，经济增长明显放缓，效益增幅大幅回落，全市生产总值没有完成1000亿元的年度目标，规模以上工业增加值、利税、利润逐月下降，财政收入增速大幅回落，特别是第四季度全部财政收入和地方一般预算收入均为负增长。这是多年来没有出现过的情况。二是产业结构不合理，节能减排任务艰巨。第一产业比重偏高，分别高于全国、全省5个和3个百分点左右，农业基础薄弱的状况没有根本改变，农民持续增收的难度加大，城乡收入差距逐渐扩大；第三产业比重在全省最低，分别低于全国、全省13个和7个百分点左右；特别是第二产业中，主导产业链条短，企业自主创新能力弱，名牌产品少，缺少立市、立县的大项目、好项目，总体竞争力不强。节能减排虽然完成了年初目标，但到目前为止，万元GDP能耗降幅还不到“十一五”目标进度的一半，今明两年任务艰巨。三是城镇化水平低，基础设施欠账较多。我市城镇化率分别低于全国、全省7个和3个百分点左右；基础设施欠账多，南北受到挤压，东西方向没有铁路和高等级公路，交通不畅。四是民生问题依然突出，社会事业还存在不少薄弱环节。城乡居民收入分别低于全省平均水平1340元和511元；受经济下滑影响，就业岗位减少，就业压力增大；教育、卫生、文化等社会事业发展仍然不快，社会保障、企业改制、食品药品安全和安全生产等民生方面，还存在许多群众反映强烈的问题。五是政府服务水平有待提高，发展环境亟需优化。少数政府部门和工作人员缺乏大局观念，服务、创新意识不强；公共权力部门化，部门权力个人化，个人权力利益化；工作作风疲沓，遇到问题推诿扯皮，办事效率低下；少数政府工作人员不作为、乱作为、吃拿卡要、违规行政等现象屡禁不止；形式主义、官僚主义和奢侈浪费、消极腐败现象仍然存在。对这些问题，我们一定要始终保持清醒的头脑，以对全市人民高度负责的使命感、责任感，认真加以解决，决不辜负全市人民的殷切期望！

　　二、2009年工作的目标要求和主要措施

　　今年是新中国成立六十周年，也是实施“十一五”规划、谋划“十二五”规划的关键一年。做好今年的政府工作，对于维护改革发展稳定大局，推进我市全面建设小康社会进程，具有十分重要的意义。

　　立足新起点，实现新跨越，我们必须科学冷静分析不断变化的发展形势，正确认识面临的机遇和挑战。当前，金融危机仍在蔓延，经济衰退仍在扩散，对我国、我市经济的影响明显显现，经济下行速度比预想的严重。我市煤炭、钢铁、水泥、建材和纺织等行业受到了较大冲击，企业投资行为更加谨慎，今年经济运行可能面临更加困难的局面。尽管存在不少困难，我们对做好今年的经济社会工作仍然充满信心。一是我国经济社会发展的基本面、所处的战略机遇期、支撑发展的基本条件没有改变；改革开放30年来，我们国家已经积累了坚实的物质基础，有效应对了历次经济波动和危机，具有驾驭市场经济、应对风险的能力；中央实施积极的财政政策和适度宽松的货币政策及保增长、扩内需的一系列措施，为我们扩大投资、推进重点项目建设提供了重要机遇。二是金融危机促使我们深刻反思粗放式发展模式的弊端，增强了加快调整结构、转变发展方式的自觉性和主动性，为我们深化改革、扩大开放、加速产业转型升级提供了战略机遇。三是经过多年来的努力，我市经济实力明显增强，产业基础更加坚实。特别是前不久召开的市委七届四次全会，提出了开创科学发展富民强市新局面的明确要求，全市广大干部群众倍受鼓舞，思想高度统一，形成了上项目、扩开放、抓升级、促调整的共识，求发展、惠民生、保和谐的愿望和热情空前高涨，这为我们应对挑战、克难攻坚、推进发展提供了强大的精神动力。因此，只要我们审时度势、把握机遇、开拓创新、迎难而上，完全有条件变压力为动力，化挑战为机遇，把各种不利影响降到最低限度，顺利渡过难关，实现我市经济社会平稳较快发展。

　　今年政府工作的总体要求是：全面贯彻落实党的十七大、十七届三中全会和省、市委七届四次全会精神，以邓小平理论和“三个代表”重要思想为指导，深入学习实践科学发展观，进一步解放思想，把“科学发展、富民强市”作为总目标，把保持经济平稳较快发展作为首要任务，把增投入、扩内需作为保增长的根本途径，把加快发展方式转变和结构调整作为保增长的主攻方向，把深化改革、扩大开放作为保增长的强大动力，把改善民生作为保增长的出发点和落脚点，创新强市、创业富民，努力实现经济社会平稳较快发展。

　　今年经济社会发展主要预期目标是：全市生产总值增长10%左右。规模以上工业增加值增长10%以上。全部财政收入增长11%，其中地方一般预算收入增长12%。全社会固定资产投资增长20%以上，其中城镇固定资产投资增长22%以上。社会消费品零售总额增长17%。出口总值增长15%左右。城镇居民人均可支配收入增长11%以上，农民人均纯收入增长7%以上。城镇新增就业4万人。万元生产总值能耗下降5.78%。COD和二氧化硫排放量分别下降6%和4%。

　　完成今年目标任务，必须以“保增长、调结构、扩内需、惠民生”为主线，重点做好以下八个方面的工作：

　　（一）努力扩大投资规模，强力推进项目建设。目前，投资仍是拉动我市经济增长的主要动力，因此，我们必须把项目建设作为保增长、促发展的“牛鼻子”，紧紧抓住、毫不放松。

　　加快重大项目建设。积极实施“8222”投资促进计划，加大项目建设力度，全年安排重点建设项目160个，总投资800亿元，力争年内完成投资230亿元。加大项目谋划力度。认真研究国家和省产业政策，紧紧围绕中央资金投向，加快谋划、筛选、包装、论证一批重大项目，完善各项前期手续，加强跑办沟通，争取列入国家投资计划。加大市本级重大项目的谋划推进力度，促进市本级经济发展。严格管好用好资金，提高资金使用效益。坚持扩大投资规模和优化投资结构并举，严格控制“两高”和产能过剩行业投资，防止低水平重复建设和盲目扩张。抓牢重大工业项目。重点抓好建滔180万吨甲醇、宁晋盐矿及盐化工、沙河金丰风电设备铸造等重大项目前期工作，争取早日开工；加快建滔50万吨醋酸、邢钢不锈钢线材技改等重大项目建设进度，力促早日投产达效。抓紧基础产业项目。争取南宫30兆瓦生物质发电项目建成发电；大力推进兴泰、任县和临城3个2×300兆瓦热电联产项目，争取列入国家专项规划；加快邢北煤田前期工作进度，争取北掌煤田开工建设；加快南水北调中线主体及配套工程建设。抓实高新技术项目。重点支持发展宁晋县和市开发区单晶硅、巨鹿县新型聚合物锂离子电池、宁晋县生物标志分子开发、临城县单螺杆技术产业化等优质项目，努力实现数量扩充型向质量提高型的转变。

　　加强项目协调调度。实行“月通报、双月调、季小结、半年一观摩、年终一评比、重大项目领导分包”等工作制度。增加项目建设在各级政府及其部门年度考核中的权重，切实把各级干部的思想和精力集中到抓项目上来。加强协调调度，认真解决征地、拆迁、环保、供电等方面的实际问题，确保项目建设顺利进行。

　　用足用好国家积极财政政策和适度宽松的货币政策。围绕国家鼓励和支持的领域，认真谋划对接项目，努力争取中央、省预算内资金和国债资金。建立和完善投融资平台，加快国有“四资”整合运作，带动社会资金，扩大融资规模。利用国家实施适度宽松的货币政策和取消商业银行信贷规模限制的时机，协调各金融机构扩大对“三农”、中小企业、基础设施、社会事业等重点领域的信贷投放，力争全年新增贷款100亿元以上。搭建银企合作平台，创造良好的金融生态环境，特别是抓好市政府与中国建设银行河北分行等金融机构间合作协议的落实，引导企业尽量多争取贷款，解决企业融资难问题。

　　（二）积极调整产业结构，加快转变发展方式。推进产业结构优化升级和发展方式转变，是提高产业整体竞争力的关键环节。因此，我们要坚持以科学发展为导向，以技术创新为先导，以节能减排为突破口，推动结构调整取得实质性进展，不断提高经济发展的质量和水平。

　　推进产业结构转型升级。对冶金及机械装备制造、煤化工、硅材料光伏等“三新”产业，积极促进加工链条向上、下游延伸，加速扩张产业规模，壮大总体实力。紧紧抓住省鼓励装备制造业做大做强的有利时机，大力整合冶金及机械装备制造产业链，倾力打造“两大基地”、“六大特色产业集群”，力争用五年时间把我市建设成为河北省乃至全国的冶金及机械装备制造业强市。积极延伸甲醇深加工、煤焦油深加工、粗苯深加工等煤怨ぁ叭蟛盗刺酢保⒄寡肪茫岣咂笠底酆闲б妗４罅Ψ⒄固裟艿绯厣卣固裟艿缯竟こ桃滴瘢蛟焱暾墓璨牧瞎夥盗刺酰銮康钟谐》缦盏哪芰Α６孕滦徒ú摹⑹称芬揭⒎闹暗取叭亍辈担酶咝录际鹾拖冉视霉ひ占涌旄脑焐恫椒ィ岣卟凡愦危嘤笠岛椭放啤?/FONT>

　　坚决打好节能减排攻坚战。节能减排作为一项硬任务, 我们要确保目标不变、标准不降、力度不减。重点抓好列入省、市“双三十”单位和30家重点耗能企业的节能减排项目，加快钢铁、电力、建材、焦炭、造纸等重点耗能、污染行业淘汰落后产能的步伐。年底前，对全市水泥企业全部实行新型干法水泥工艺。继续开展“城镇污水垃圾处理设施建设三年攻坚行动”，确保七里河和各县（市）污水处理厂全部建成投入运行，建成垃圾处理厂12座，市医疗垃圾处置中心建成投运。继续加强市区大气综合治理，空气质量二级及好于二级天数稳定在320天以上。强化目标考核，对节能减排工作进展缓慢、逾期完不成目标任务的单位，严格执行问责制和“一票否决”，让我们牛城的天更蓝、水更清、空气更清新。

　　促进民营经济加快发展。认真落实鼓励民营企业发展的各项优惠政策，加强对重点民营企业、成长型中小企业和重点特色产业集群的支持力度。鼓励民营企业增加研发投入，加强品牌建设，实现转型升级，向专、新、特、精方向发展，提高市场竞争力，发挥其在促进就业方面的特殊作用。建立和完善中小企业信用担保体系，提高担保能力。加强创业辅导基地建设，进一步推动和鼓励全民创业。重视培养壮大现代企业家队伍，着力帮助他们解决在发展中遇到的困难和问题。

　　确保工业经济平稳运行。加强工业运行调节，完善监测预警体系，全力帮助协调解决企业生产经营中遇到的困难和问题。加强煤电油运和重要原材料的组织协调、供需衔接，确保重点企业生产需求。引导企业积极应对市场变化，鼓励行业龙头企业、优势企业兼并重组落后企业、困难企业；鼓励优势企业强强联合，提高产业集中度和规模效益；鼓励关联产业、上下游企业联合重组，实现一体化经营，增强抗风险能力；支持企业跨区域联合重组，增强整体实力；引导外贸出口企业调整产品结构，拓展国内市场。

　　积极实施名牌战略。以高端、精品、专业化、深加工为方向，引导企业争创驰名商标和名牌产品，打造一批具有自主知识产权和较强市场竞争力的知名品牌。重点做好宁晋永进电缆、隆尧远大阀门和晶牛微晶玻璃等中国驰名商标申报工作，力争新增省以上知名品牌15个以上。

　　（三）深入推进三年大变样工作，致力构建大城市新格局。深入开展“三年大变样”工作，既是扩内需、增投入、保增长的有效手段，更是完善城市功能、改善居民生活环境、促进经济社会平稳较快发展的战略举措。因此，我们要以这项工作为载体，着力打造“五大亮点”。

　　打造“一城五星”大城市框架。突出中心城市的首位带动作用，积极推进市区与环城五县（市）一体化战略，加快市区到五个卫星城之间的快速公路建设，形成10分钟交通圈。谋划实施功能分区和园区布局，吸纳市内外经济要素向交通圈内的快速公路沿线聚集，向产业园区聚集，向中心城市大框架内聚集。到2020年，逐步建成人口260万以上的大城市框架，使之成为拉动邢台经济发展、推进城镇化的强大引擎。同时，大力推进清河、宁晋两大次中心城市和各县城及重点镇建设，努力形成大中小城市协调互动发展的格局。

　　打造百里太行工业经济带。加快完善基础设施，建设提升沿百里带的旭阳工业园、官庄大孟工业园、羊范工业园、清修岗工业园等园区，加大开放招商力度，吸引一批重大项目入驻园区。把园区与城镇规划建设结合起来，合理进行功能定位和划分，通过工业项目的带动，推进城镇化建设，逐步把百里太行工业经济带建成聚集项目、聚集财富、聚集人口的城镇连绵区。

　　打造西联东出、贯通南北的交通枢纽。围绕改变我市当前“东西不畅、南北挤压”的被动局面，加快推进一批重大交通干线建设。尽快建成东西方向的快速交通通道，年内开工建设邯（邢）黄铁路、邢汾高速、邢临高速卫运河大桥；加大邢和铁路前期工作力度；邢港高速争取列入省建设规划，带动环城高速建设，实现“四小时出海，半小时进山”目标。南北方向加快石武客运专线、大广高速公路施工进度，开工建设京港澳高速改扩建项目，进一步提高运输能力，尽快使我市成为河北南部和中原地区的物资集散、要素汇集又一交通枢纽，提高区域竞争力，为我市经济社会发展提供强力支撑。

　　打造宜居宜业新牛城。市区全年投资145亿元，实施一批重点城建工程，完善城市功能，提升城市品位，建成人民工作生活的福地、客商投资置业的宝地。按照“继续拆、突出建、强化管”的要求，推进城镇建设。拆违拆迁，拆出空间、拆出土地。重点向小街小巷延伸，向城中村和矮旧建筑延伸，向铁路沿线延伸，全年全市完成500万平方米以上，其中市区完成200万平方米以上，到年底累计拆迁量达到既有建筑面积的15%以上。城镇建设，建出亮点、建出精品。加快七里河综合治理进度，按照“政府主导、市场运作”的原则，加大投资力度，在基本完成河道治理的基础上，建设“两园一带四桥一道”，启动土地开发建设项目，尽早取得社会、经济和生态效益。围绕建设“九纵九横加三环”的城市道路交通目标，大力实施城市主干道外延和市区8个出市口改造，打通市区主要街道的断头路，完成邢州路、钢铁路等10条街道景观整治，启动郭守敬大道、百泉大道、东环路、滨江路建设改造工程，打造通畅靓丽、整洁有序的城市交通路网。引导开发商建设适销对路住房，满足不同消费群体的需求。城市管理，管出品位、管出形象。统筹整合城管、公安、交通、建设、环保、消防等职能部门的公共管理资源，建立网格化管理模式，提高应急处置能力。按照科学精细、文明执法的要求，狠抓市容市貌整治，塑造环境整洁、居住舒适、生态文明的城市新形象。

　　打造农村新民居示范村。坚持规划先行、分类缘肌⒁缘愦妗⒑托彻步ǖ脑颍韧椎亟ㄉ枰慌┐逍旅窬邮痉洞澹昴谌辛φ蛟?00个示范村，形成示范带动效应，促进全市农民居住环境的改善，逐步达到城市居民的居住条件。逐村搞好村庄建设规划，做到无规不建。积极开展“空心村”治理和村边闲散土地开发，整合并集中使用农村自来水、道路等项目资金，解决建设土地和资金问题。

　　（四）切实抓好“三农”工作，努力促进农民增收。农民增收是“三农”工作的重中之重。我们要认真落实市委七届四次全会上通过的《关于做好农村改革发展十项重点工作的意见》，加快建设现代农业，力争农业效益和农民收入有较大幅度的提高。

　　围绕提高效益调结构。稳定粮食播种面积，优化品种结构，将全市粮食产量稳定在80亿斤以上。进一步发展壮大畜牧、林果、蔬菜三大主导产业，使其占大农业的比重达到65%以上。畜牧业，打造黑龙港流域奶业发展中心，大力推进奶牛标准化养殖，优质高产奶牛存栏达到8.5万头；以凯特300万只品牌蛋鸡为龙头，形成沙河、南和等6个蛋鸡标准化养殖基地；建成年出栏10万头大型猪场1个、万头以上10个、千头以上50个。林果业，精心培育富岗、浆水苹果，绿岭薄皮核桃，太行板栗和新河、南宫大枣等一批知名品牌；发展农林复合经营，加快东部平原林网建设；实施太行山绿化工程和绿色通道建设工程，抓好小流域综合治理，利用两年时间基本完成丘陵区绿化工作。蔬菜业，着力抓好环市区精品蔬菜生产带、沿106国道高效蔬菜生产带、沿308国道食用菌生产带和西部山区错季蔬菜生产区“三带一区”优势蔬菜生产。健全农产品质量安全监管体系，防止不合格农产品进入市场。同时，加强动植物重大病虫害和疫病防控，切实搞好防汛、防火、防雹等工作，促进农业农村经济健康发展。

　　大力推进农业产业化经营。我们要以抓工业的思路抓农业，以经营服务业的理念经营农业，促进第一产业向第二产业、第三产业延伸。第一产业向第二产业延伸就是要抓好农业产业化龙头，着力发展农产品精深加工，实行项目、基地、农业经济合作组织、园区、产业整体推进，规范生产标准，突出品牌建设，扩大生产规模，在提高农产品附加值和市场占有率上下功夫。重点抓好豪达牧业肉鸡、南宫燕丰食品等10个超亿元大项目，力争年内完成投资50亿元，新增销售收入超5000万元龙头企业5户，全市农业产业化经营率达到61%。第一产业向第三产业延伸就是要抓市场、中介组织等农业服务型龙头，提高农产品市场化和农民组织化程度。

　　加强农业农村基础设施建设。落实好中央、省关于加大对农村基础设施建设投入力度的政策要求，市、县两级地方财政对农业投入增长高于经常性收入增幅。启动列入省粮食生产核心区县的优质粮产业工程及标准良田建设；加快重大农田水利工程建设，全面完成临城水库除险加固工程，争取青山水库项目通过国家发改委立项；继续实施土地治理、节水灌溉等农业项目。深入开展“乡村洁绿工程”，完成10个省级乡村清洁循环利用工程示范村建设。完成投资2亿元，推动农村道路改造升级和村级道路连网。加大农村信息网络化建设力度，提高互联网、有线电视、通讯等信息网络覆盖率。

　　健全农业农村服务体系。结合农业科技特派员制度，加快基层农技推广体系建设，实施“一村一名大学生”计划，提高农民科技素质，做到一户一个明白人；大力普及“两晚”、节水、配方施肥等先进适用技术；认真落实农机具补贴政策，鼓励农民购置、使用先进农业机械，提高耕作效率；积极培育、引进、扩繁一批新优特品种，建设高产、优质、高效、生态、安全农业。大力发展村镇银行，抓好小额贷款担保公司试点，解决农民贷款难问题。

　　促进农村劳动力转移。加强对农村劳动力的职业技能培训，着力培养一大批具有较高文化素质和就业技能的新型农民，促进农村劳动力向非农产业和城镇转移。当前，切实加强对农民工返乡创业就业的支持，搞好对他们的就业能力培训，发挥他们的优势，鼓励他们带头创业，投身农村基础设施建设和中小企业发展。

　　（五）加快发展第三产业，尽快扭转滞后局面。服务业是扩大居民消费、提升生活质量、推动经济发展的重要着力点，我们一定要突出重点，积极推进，大力增强服务业对经济增长的拉动作用。

　　积极培育现代服务业。优先发展现代物流、信息服务、研发设计、金融保险等生产性服务业，为加速城镇化、工业化进程提供强大支撑。加快推进好望角物流、河北北方粮油物流、龙岗物流、烟草物流等项目建设，努力打造物流龙头企业。同时，积极发展养老、托幼等新兴服务业，大力发展投资咨询、资产评估等咨询服务业以及会展、法律、会计等中介服务业。

　　构建城乡商贸流通体系。深入实施“万村千乡”和“双百”市场工程，全市累计建设3000家农家店，信息化建设比例达到50%，商品统一采购或连锁配送率达到50%以上，覆盖所有乡镇和50%以上的行政村；“双百”市场工程建设实现突破，多个农产品批发市场列入省级标准化农产品批发市场，强力打造农村商贸流通新体系。支持服务业重点项目建设，城镇黄金地段优先用于发展服务业。中心城区在做大做强和创新提升传统商贸服务业的基础上，吸引新项目，增加新设施，发展新业态，培育壮大家乐园、银座、新亚集团等龙头企业。城区周边规划建设2个大型批发市场，努力打造布局合理、快捷方便、满足市民需要的城区现代流通新体系。

　　积极拓展城乡消费市场。落实好鼓励消费的财税、金融等政策措施，改善居民的消费预期。进一步稳定扩大住房和家电、农机等大宗消费，培育文化、体育等新的消费热点。引导市民转变消费方式，倡导健康饮食习惯，市区启动实施“大众厨房工程”，中心城区早餐车总数达到100辆，大众餐厅达到20个，大众快餐示范店达到20家。组织好“家电下乡”工作，落实国家补贴政策，推动商业连锁经营向农村延伸，努力提高我市农村居民消费水平。

　　大力发展旅游业。精心规划，整合资源，突出特色，着力打造“山水太行、守敬故里”杂挝幕放啤４罅Ψ⒄股角糜危嘀粕角糜巫芴骞婊胂晗腹婊研咸ㄏ亍⑸澈印⒘俪恰⒛谇?个县（市）的旅游资源整合成各具特色、分工合作的旅游景点。推广岗底、前南峪等地的成功开发模式，搞好西部百里太行生态环境建设。抓紧谋划实施连接太行山区精品景区的主干道路建设，新建和筹划一批较高档次的旅游饭店、农家旅馆和游乐设施，构建起吃住游一体化的综合服务体系。同时，加大宣传促销力度，不断提高邢台旅游产品知名度。

　　（六）继续深化重点领域改革，全面提高对外开放水平。改革开放是我们应对当前困难、促进经济增长和推进科学发展的保障。因此，我们要加大改革的力度和深度，提升开放的层次和水平。

　　深入推进重点领域改革。加快企业改革，贯彻实施《企业国有资产法》，进一步理顺经营性国有资产监管体系；深化企业内部改革，完善法人治理结构，建立规范的现代企业制度；整合中小钢铁、煤炭企业和地方矿产资源；推进中小型国有企业改制和劣势企业退出。不断深化农村改革。稳定和完善农村基本经营制度，按照依法、自愿、有偿的原则，稳步推进农村土地承包经营权流转。以南和、威县、清河、巨鹿等县为试点，建立乡级土地流转服务平台，发展多种形式的适度规模经营，促进农业规模生产和劳动生产率的提高。同时，努力壮大农村集体经济，积极推进农村综合改革、集体林权制度改革和其他改革。深化财政体制改革，积极培植财源，依法强化征管；优化财政收入结构，做大做强市本级财政收入；严格控制和压缩一般性支出，集中有限财力办大事。加快金融及投融资体系改革步伐，推进国有股份制银行改革，支持我市商业银行做大做强，积极引进华夏、民生等股份制银行入驻邢台。同时，加快推进价格、收费等改革。

　　加大开放招商力度。用足用好省关于产业聚集区建设的政策，大力支持产业基础条件和发展前景好的产业聚集区加快发展，争取列入省级产业聚集区。牢固树立工业强市的理念，瞄准立市、立县的大项目，盯住世界500强和国内有实力的大集团、大企业，定向跟踪，主动对接，寻求合作。特别是加强与已建立合作关系的中钢、建滔、中煤、中盐等大企业的沟通联系，进一步拓宽合作领域，努力扩大合作规模；加强对协议利用外资500万美元以上项目的跟踪调度，提高合同履约率和外资到位率。充分利用国家出口退税政策，调整优化出口产品结构，努力提高出口对经济增长的拉动作用。支持晶龙集团、中钢邢机等出口大户进一步扩大出口规模，争取再建成1——2个省级出口基地。

　　（七）精心实施民生工程，切实改善人民生活。关注民生是科学发展、以人为本的体现。越是经济困难的时候，越要高度关注民生、重视民生、改善民生，尤其是困难群体的民生问题。今年我们要重点实施十项“民生工程”。①就业再就业工程。年内为大中专院校毕业生提供就业岗位2万个；城镇下岗失业人员实现再就业1.8万人；转移农村富余劳动力22万人（次）以上，劳务输出10万人（次）以上。②国有企业职工安置工程。多渠道筹集资金，加大力度，创新方法，力争全部完成市属工业企业的改制工作，安置所属职工1.7万余人；力争全部完成市属商贸流通企业改制工作，安置所属职工1万余人。③社会保障工程。大力推进社会保险扩面征缴，城镇基本养老保险覆盖人数达到43万人；医疗、生育、失业、工伤保险参保人数分别达到45万、20万、33万、30万人。今年企业退休人员基本养老保险金再提高10%以上。指导企业在效益提高的前提下，适度提高职工工资，稳步推进事业单位工资改革。启动城镇居民医疗保险试点工作，确保首次参保率达到50%以上；在校大学生全部纳入城镇居民医保范围。提高城乡居民最低生活保障标准，一步到位达到全省平均水平，并建立同步增长机制。进一步加快星级敬老院建设进度，确保每个县至少建成一所星级敬老院，年底前五保户集中供养率达到50%以上，同时积极探索五保老人供养新途径。继续推进新型农村合作医疗工作，全市参合率达到90%以上，参合农民补偿封顶线由2万元提高到3万元。④住房保障工程。年内开工建设住房面积200万平方米，其中普通商品房和保障性住房不少于150万平方米；市区继续补贴经济适用住房家庭1000户，补贴资金3000万元；廉租住房做到应保尽保，建设廉租住房1477套，补贴2600户，实物配租800套。按照适用、经济、低价、自愿的原则，推进农村中小学教师住房建设，今明两年基本解决农村教师城镇住房问题。改善、改建4个旧住宅区，启动28个城中村改造和一批旧城区、危旧房改造；实施采煤沉陷区民房搬迁维修工程，全年完成搬迁6173户，加固维修3404户。⑤农村生态家园富民工程。按照“一建三改”模式，搞好“三位一体”，新建户用沼气池3万个以上，建成养殖小区和联户沼气设施10处、规模化养殖场大中型沼气工程6处。⑥农村饮水安全工程。大力推广联村供水模式，解决32万农村人口的饮水安全问题。⑦农村教育基础设施改建提升工程。改造农村中小学80所，新建或改扩建校舍面积8万平方米，消除危房面积1万平方米，同时，进一步巩固“温暖工程”成效。⑧农村基层文化站建设工程。争取74个乡镇综合文化站开工建设，建成32个乡镇综合文化站。⑨市区生活环境改善工程。对市区所有的街道全部实施亮化，搞好72条街道、21个广场游园和3条河道的绿化；新建22个小型路边游园、25个小片林、20个小便民市场、50处小停车场、400个出租车升降站、30个公共厕所和若干垃圾收集点；新增民用燃气用户1万户，集中供热面积230万平方米。⑩平安邢台建设工程。积极做好群众来信来访工作，搞好矛盾排查化解，基层矛盾化解率达到95%以上；强化社会治安综合治理，严厉打击各种刑事犯罪活动，社会治安群众满意率达到98%以上，确保政治安定、社会稳定。

　　为民多办实事、好事是政府的天职，我们将尽职尽责、尽心尽力，把这十项民生工程一件一件地抓实、抓好，真正使发展成果体现在人民生活质量的提高上，体现在人民群众的笑脸上！

　　（八）大力繁荣社会事业，积极促进协调发展。发展社会事业是科学发展的重要体现，我们要把社会事业摆在更加重要的位置来抓。优先发展教育事业，加强中等职业教育基础能力建设，支持市农业学校等5所学校建设实训基地，市区和皇寺两个职教园区建设取得实质性进展；全市高中段毛入学率达到80%以上。提高教师队伍素质，努力推出一批名师、名校和名学科。加快推进科技创新和成果转化应用，支持企业建设技术研发中心，加强与大专院校、科研院所的联合协作。提高医疗卫生服务水平，争取全部完成乡镇卫生院的改扩建任务，新建村卫生室42个；加大对乡镇卫生人员的培训力度，积极推进农村卫生服务一体化试点工作；完善城市社区卫生服务体系，加快社区卫生服务中心建设步伐，街道覆盖率达到95%以上。加快文化事业发展，加快市图书馆迁建和广电中心建设，启动博物馆和全民健身广场建设；组织好各类文化活动，积极推进特色文化产业发展。进一步强化食品药品安全，从生产、销售、消费等各个环节抓起，健全安全监管体系，深入整顿和规范市场秩序，严厉打击无照经营、非法生产、制售假冒伪劣食品药品等违法行为。把今年作为安全生产工作的“攻坚年”，进一步完善监管措施，着力打好煤矿整合关闭和瓦斯治理、尾矿库治理、危化行业整治、打击非法生产经营活动四个攻坚战，对存在严重安全生产隐患的企业，该整顿的彻底整顿，该关闭的坚决关闭，决不能留下死角和隐患。落实安全责任，严格执行行政问责制，坚决惩处安全事故涉及的失职、渎职和各种腐败行为。深入开展“双拥”共建活动，支持国防后备力量建设。加强人口和计划生育工作，稳定低生育水平。同时，做好体育广电、民族宗教、外事侨务、妇女儿童、应急管理、人防、气象等各项工作。

　　三、切实加强政府自身建设

　　在当前复杂多变的严峻形势下，全面完成今年各项目标任务，是对各级政府的严峻考验。我们必须认真贯彻落实市委七届四次全会精神，以更大的力度解放思想、以更大的气魄推进科学发展、以更开阔的思路推进改革开放、以更有力的措施转变工作作风，努力开创政府工作的新局面。

　　第一，以更高标准推动科学发展。深入开展学习实践科学发展观活动，自觉把邢台发展放在全省乃至全国的大局中进行比较分析，增强加快发展的压力感、责任感和紧迫感。紧紧扭住发展第一要务不动摇,把最主要的精力用来谋划发展，把最主要的职能用来服务发展，把最主要的力量用来推动发展。我们要针对落后指标，认真分析落后原因，紧紧盯住省内和中原协作区13市中的先进市，制定出切实可行的追赶计划，在一个一个指标的追赶中，在一点一点新的进步中，努力实现我市经济社会全面协调可持续发展。

　　第二，加快转变政府职能。强化社会管理和公共服务职能，集中更多的精力和资源为群众、为企业、为客商搞好服务。深入推进行政审批制度改革，缩短时限、精简程序、提高效率。完善公共决策机制，提高行政决策的科学性、民主性。贯彻实施政府信息公开条例，促进政府工作公正、公平、公开、便民。进一步完善行政管理机制，努力解决好职能交叉以及政府越位、缺位、错位等问题。积极推进行政管理体制改革，切实做到简政放权，把属于市场的职能还给市场，把属于政府的职能高效履行好。

　　第三，着力改进工作作风。大力倡导市委七届四次全会上提出的“五办”作风，深入开展“作风建设年”活动，着力解决不尽职责、服务差，不讲标准、效率低，不守纪律、乱作为等问题。把抓落实作为改进作风的核心，强化考核追责任，盯住目标要结果，把该做的事一定做好，决不让决策部署因执行不力而落空。建立改进工作作风的监督机制和长效工作机制，以新食尚公司反映的发展环境问题为突破口，深查问题，举一反三，严格整改。目前，市政府各部门正在媒体上陆续作出公开承诺，我们真诚欢迎广大群众监督批评。市监察局、市优化办、市政府督查室等部门要加强监督，严格检查。对这些措施我们一定要落到实处，向人民作出的承诺我们一定要兑现，对影响发展环境的任何单位和个人，一经发现，严肃处理，决不姑息。

　　第四，全面实施依法行政。加大依法行政工作力度，进一步规范行政行为，加强行政执法队伍建设和对执法活动的监督，全面落实行政执法责任制。自觉接受人大及其常委会的监督，坚持定期向人大常委会报告工作，向政协通报情况，认真接受政协委员的民主监督，加强与政协的工作沟通和协商，切实做好人大代表建议和政协提案的办理工作。广泛听取民主党派、工商联、无党派人士和各人民团体的意见，发挥好新闻舆论和社会公众监督的作用，使政府依法、有序、高效地为人民工作好、服务好。

　　第五，切实加强廉政建设。大力弘扬谦虚谨慎、艰苦奋斗的精神，牢固树立过紧日子的思想，今年全市公用经费一律压减10%，严格控制公务购车用车、会议接待等费用支出；严格控制出国考察；严格控制机关事业单位楼堂馆所建设，严禁超面积、超标准建设和装修。坚决反对滥用职权、损公肥私、弄虚作假、铺张浪费、浮华奢侈等行为，大力纠正公款吃喝、公车私用等不正之风。坚持依法行政、从严治政，自觉把行政权力的运行置于人民群众监督之下；坚持民主评议、效能监察，切实加强对权力运行的监督和制约；坚持标本兼治、注重治本，着力从源头上预防和治理腐败，为邢台人民营造一片公平正义、风清气正的天空。

　　各位代表，全心全意为人民服务是我们的职责。加强政府自身建设，归根到底就是要把人民的利益放在首位，做到权为民所用，情为民所系，利为民所谋，为人民群众办好事、做实事、谋利益、增福祉。我们一定要切实做到为民、务实、清廉，努力建设勤政廉政的政府、高效负责的政府、让人民满意的政府！

　　各位代表，今年的形势复杂而严峻，任务艰巨而光荣。让我们紧密团结在以胡锦涛同志为总书记的党中央周围，在省委、省政府和市委的正确领导下，围绕“科学发展、富民强市”目标，同心同德、开拓奋进，迎难而上、真抓实干，全面完成今年的各项目标任务，以优异成绩向新中国成立六十周年献礼！
